# The effect of genetic robustness on evolvability in digital organisms

**DOI:** 10.1186/1471-2148-8-284

**Published:** 2008-10-14

**Authors:** Santiago F Elena, Rafael Sanjuán

**Affiliations:** 1 Instituto de Biología Molecular y Celular de Plantas, CSIC-UPV, 46022 València, Spain; 2 The Santa Fe Institute, Santa Fe, NM 87501, USA; 3 Institut Cavanilles de Biodiversitat i Biologia Evolutiva, Universitat de València, P.O. Box 22085, 46071 València, Spain; 4 Departament de Genètica, Universitat de València, Burjassot, 46100 València, Spain

## Abstract

**Background:**

Recent work has revealed that many biological systems keep functioning in the face of mutations and therefore can be considered genetically robust. However, several issues related to robustness remain poorly understood, such as its implications for evolvability (the ability to produce adaptive evolutionary innovations).

**Results:**

Here, we use the Avida digital evolution platform to explore the effects of genetic robustness on evolvability. First, we obtained digital organisms with varying levels of robustness by evolving them under combinations of mutation rates and population sizes previously shown to select for different levels of robustness. Then, we assessed the ability of these organisms to adapt to novel environments in a variety of experimental conditions. The data consistently support that, for simple environments, genetic robustness fosters long-term evolvability, whereas, in the short-term, robustness is not beneficial for evolvability but may even be a counterproductive trait. For more complex environments, however, results are less conclusive.

**Conclusion:**

The finding that the effect of robustness on evolvability is time-dependent is compatible with previous results obtained using RNA folding algorithms and transcriptional regulation models. A likely scenario is that, in the short-term, genetic robustness hampers evolvability because it reduces the intensity of selection, but that, in the long-term, relaxed selection facilitates the accumulation of genetic diversity and thus, promotes evolutionary innovation.

## Background

Evidence has accumulated during recent years suggesting that organisms can maintain their performance in the face of a broad range of perturbations [1,2]. This includes the tolerance of proteins to amino acid replacements [3], the ability of genetic networks to withstand alterations to its parts [4], the stability of cellular processes to the stochastic variations of gene expression levels [5], or the resilience of embryonic development to environmental or genetic changes [6]. In general, the term 'robustness' is used to describe this kind of behavior and, when mutations are the cause of perturbation, the term 'genetic robustness' or 'mutational robustness' is used. Many issues related to genetic robustness remain unresolved. For example, asserting that elevated robustness is a fundamental property of living organisms is problematic because we often ignore what normal robustness should be [7]. Still, we can try to identify the genetic and ecological factors associated with differences in robustness between species or genotypes [8,9]. Also, it remains unclear whether the evolutionary transition to a robust state occurs as a direct product of selection [10-13] or merely as a by-product of selection acting on correlated traits [14-16].

A system is said to be evolvable if it can be modified through genetic change in a way that enhances survival and reproduction. For natural selection to act, the system must show heritable phenotypic variation. Yet, genetic robustness implies that the system produces little phenotypic variation in response to genetic variation. Therefore, robustness might limit evolutionary optimization and innovation [17]. In this vein, theoretical work has postulated that buffering mechanisms can lead to maladaptation compared to what would be achieved in the absence of these mechanisms [18]. Also, the analysis of gene expression noise in yeast suggests that noise control may indirectly increase mutational robustness, which might in turn hamper evolvability at the level of gene expression [19]. On the other hand, genetic robustness facilitates the accumulation of neutral or nearly neutral variation by relaxing the intensity of natural selection. This accumulated diversity can become visible to selection upon changes in the environment or the genetic background, and thus be the source of evolutionary innovation. Computer simulations on simple population genetics models predict that genetic robustness can sometimes facilitate access to new adaptive peaks provided that occasional failures of robustness mechanisms occur [20]. The view that robustness can foster evolvability has also been supported by lattice protein models and PCR-based mutagenesis experiments showing that protein variants with increased thermodynamic stability have increased genetic robustness and are more likely to evolve new catalytic capabilities [21].

Here, we address the relationship between robustness and evolvability by directly comparing the ability of genotypes with different degrees of robustness to adapt to novel environments. At present, it is difficult to envisage such experiments with natural organisms. Current work in this area is mainly focused on the characterization of robustness in different species or genotypes from the same species [22-24]. Instead, we perform our experiments using digital organisms. Digital organisms are self-replicating computer programs that inhabit a virtual world where they reproduce, mutate, compete for resources, and evolve [25]. Since digital and natural organisms evolve under the same fundamental rules, the evolution of digital organisms should be informative about the natural world. It must be noted, however, that the particular physicochemical properties of natural systems are not always captured *in silico*, despite the potentially important role of these properties in evolution. On the other hand, the use of digital organisms offers several advantages: first, experiments can be performed on scales that are beyond reach with any biological entity; second, the study of evolution with digital organisms allows one to collect extremely precise information about the evolutionary process; third, one can easily perform certain genetic manipulations that would be exceedingly laborious on natural organisms.

Using the Avida platform [26], we first obtained digital organisms with varying levels of genetic robustness, as previously described [27]. We then evaluated the ability of these organisms to adapt to new environments. The results suggest that, in a simple environment, genetic robustness retards the first adaptive steps but clearly fosters long-term evolvability. Consequently, at any given time point, whether robustness promotes evolvability depends on the rate of adaptation and thus, indirectly, on the mutation rate, but also on the complexity of the environment. We argue that, in the short-term, genetic robustness can slow down adaptive evolution by relaxing the intensity natural selection. In the long-term, however, it can favor the accumulation of genetic variation and thus provide access to more of the fitness landscape.

## Results

### Preliminary experiment: evolution of genotypes with different levels of genetic robustness

We evolved the Avida default organism under different regimes previously shown to favor the divergence of robustness [27]. Nine regimes, resulting from the combination of three genomic mutation rates (0.02, 0.2 and 2) and three population sizes (10^2^, 10^3 ^and 5 × 10^4^) were tested, and 20 independent lineages were seeded for each regime, yielding 180 lineages in total. Organisms were maintained for 10^5 ^updates in an environment in which four logic tasks (the digital equivalent to metabolic resources) were rewarded (NOT, AND, OR, and NOR). At the end of the evolution experiment, the most frequent organism was isolated from each lineage and its adaptation to the new environment was judged from its ability to perform these four tasks, a requirement which was met by 62 of the 180 evolved organisms. Organisms not performing the four tasks were discarded from subsequent analyses. The use of organisms homogeneous with respect to their ability to perform tasks ensured that none of them would be a priori better predisposed than others to learn new tasks.

The robustness of these organisms was computed and an analysis of variance showed that, consistent with previous work [27,28], high mutation rates tended to favor the evolution of robustness (*P *< 0.001), whereas population size had no direct effect on robustness (*P *= 0.818), but and indirect one conditional on mutation rate (interaction term, *P *< 0.001). We also observed a weak negative correlation between robustness and log fitness (Spearman's *ρ *= -0.270, 60 d.f., *P *= 0.034), indicating that robustness paid some fitness cost, an observation which is also in line with theoretical considerations [8] and previous experiments [22,24,28]. As expected, robustness positively correlated with the fraction of selectively neutral mutations (*ρ *= 0.899, 60 d.f., *P *< 0.001) and negatively correlated with the magnitude of deleterious fitness effects (*ρ *= -0.376, 60 d.f., *P *= 0.003). Interestingly, the fraction of beneficial mutations also increased with robustness (*ρ *= 0.626, 60 d.f., *P *< 0.001). This later association was not a trivial consequence of the fact that more robust genotypes were less fit, because the correlation held after controlling for log fitness (partial correlation: *r *= 0.564, 59 d.f., *P *< 0.001). Finally, the magnitude of beneficial mutations did not depend on robustness after controlling for fitness (partial correlation: *r *= -0.140, 59 d.f., *P *= 0.282).

### Analysis of the genotypes showing the largest and smallest robustness values

We first performed a detailed analysis of the relationship between robustness and evolvability focusing on the two organisms with the most extreme robustness values from the above experiments, hereafter denoted *R *(robust) and *F *(fragile) (Table 1). *R *and *F *showed similar absolute fitness values, which rules out possible differences in evolvability due to differences in starting fitness values (relative fitness values are reported hereafter, but similar results were obtained with absolute fitness values). We will later assess whether the results obtained with this pair can be generalized to the rest of organisms.

**Table 1:** The two organisms with the most extreme genetic robustness values obtained from the preliminary experiment

			Percentage			Evolutionary history	
			
Organism	Fitness	$\bar{s_{d}}$	Deleterious	Neutral	Beneficial	*U*	*N*
*F*	417.19	-0.746	89.12% (0.833)	10.36%	0.52% (0.005)	0.2	5 × 10^4^
*R*	466.75	-0.377	56.84% (0.641)	39.88%	3.28% (0.005)	2	5 × 10^4^

The signed average selection coefficient excluding beneficial mutations, $\bar{s_{d}}$, was used to quantify robustness. Fitness and the percent of deleterious, neutral and beneficial mutations are shown for each organism. Numbers in parenthesis indicate the average magnitude of mutational effects. The genomic mutation rate (*U*) and the population size (*N*) under which each organism evolved in the preliminary experiment are also shown.

We placed *R *and *F *in a novel environment in which four new tasks (NAND, ORN, ANDN, and XOR) were rewarded in addition to the four original ones (for a total of 8 tasks rewarded). Differences in mutational effects held in the new environment (*F*: 80.84% deleterious, 18.84% neutral, 0.32% beneficial; *R*: 52.84% deleterious, 40.52% neutral, and 6.64% beneficial; χ^2 ^= 20.913, 2 d.f., *P *< 0.001). We seeded 50 independent lineages of each *R *and *F *and let them evolve for 1000 updates at a population size of 10^4 ^(this size was used in all subsequent experiments). Given that *F *and *R *had been previously evolved at different mutation rates (Table 1), we explored various genomic mutation rates (*U*), ranging 0.03 – 3. Also, to evaluate the stability of robustness, we recalculated it at the end of the run for each of the 50 populations evolved at *U *= 0.3. Even though genotypes evolved from *R *lost 24.38% of their robustness and those evolved from *F *increased in robustness by 20.09%, differences between the two groups were still highly significant (Wilcoxon's signed ranks test: *P *< 0.001).

**Figure 1:**
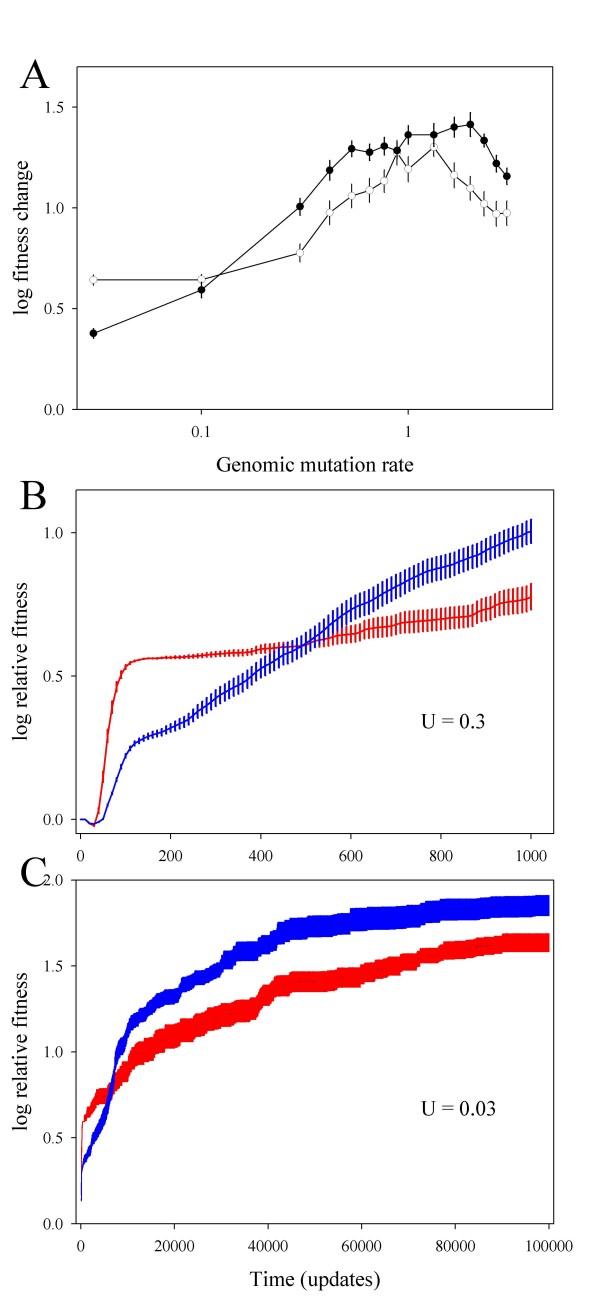
**A) Rate of adaptation to a novel 8-task environment as a function of mutation rate, for fragile *F *(white) and robust *R *(black) genotypes.** Adaptation was measured as the difference in log fitness of the evolved and ancestral organisms. Fitness was first averaged over all organisms in a population, then log transformed, then averaged over the 50 replicate lineages for each mutation rate. Bars represent standard errors of the mean. B) and C) Fitness trajectories for lineages evolved from *F *(red) and *R *(blue)for two different mutation rates and timescales.

For most mutation rates tested, *R *was more evolvable than *F *(Fig. 1A), and the difference increased with mutation rate (*ρ *= 0.521, 12 d.f., *P *= 0.046). Indeed, *F *only showed a better ability to adapt for *U *≤ 0.1. At face value, this could lead one to conclude that the benefit of robustness was directly dependent on the mutation rate. However, within the explored parameter range, the rate of evolution increased with mutation rate (Fig. 1A). Therefore, it is possible that robustness conferred an adaptive advantage only in the long-term, and that such advantage would appear to be greater at higher mutation rates. Two observations clearly supported to this possibility. First, as shown in Fig. 1B for *U *= 0.3, *R *was less evolvable in the short-term (update < 500), whereas in the long-term (update > 500), the situation was reversed. The same pattern was observed for mutation rates within the range 0.3 – 3 (not shown). Second, at low mutation rates (*U *≤ 0.1), the short-term fitness advantage of *F *was lost after sufficiently long evolutionary times. For instance, for *U *= 0.03, *R *evolved higher fitness than *F *beyond update 6500 (Fig. 1C).

**Figure 2:**
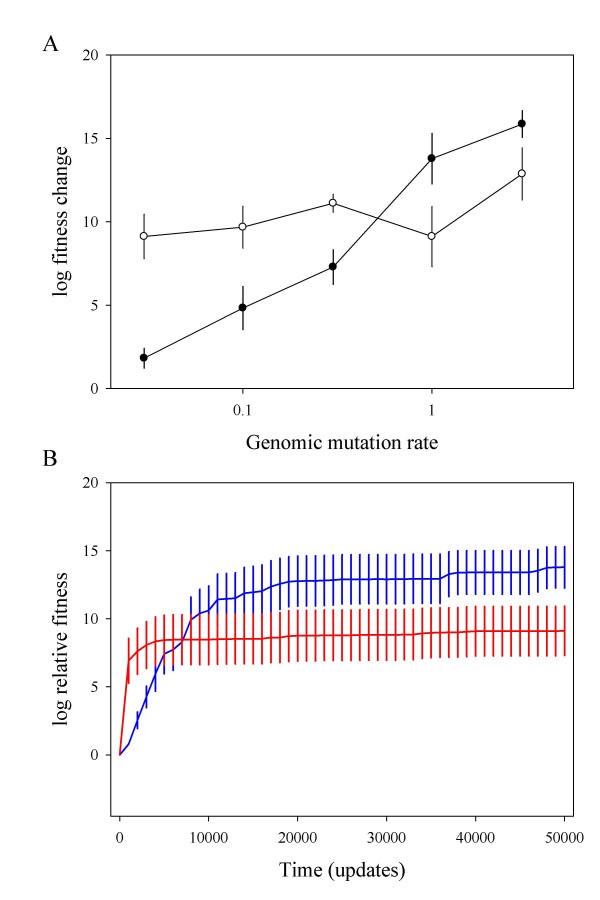
**A) Rate of adaptation as a function of mutation rate for a more complex, 77-task, environment (white: fragile genotype *F*; black: robust genotype *R*).** B) Fitness versus time for lineages evolved from *F *(red) and *R *(blue) in this environment at a mutation rate *U *= 1. Fitness was calculated in the same manner as in Fig. 1.

We then performed similar experiments in another, more complex environment, defined by 73 new rewarded tasks (a 77-task environment). Differences in mutational effects between *F *and *R *held in this new environment (*F*: 76.00% deleterious, 19.96% neutral and 4.04% beneficial; *R*: 50.52% deleterious, 42.44% neutral and 7.04% beneficial mutations; χ^2 ^= 14.042, 2 d.f., *P *< 0.001). The frequencies of deleterious, neutral, and beneficial mutations varied slightly between the 8- and 77-task environments, but these differences were not statistically significant (*F*: χ^2 ^= 2.976, 2 d.f., *P *= 0.226; *R*: χ^2 ^= 0.080, 2 d.f., *P *= 0.961). We seeded 50 independent lineages of *F *and *R *in this complex environment, using genomic mutation rates ranging 0.03 – 3. For all mutation rates tested, lineages derived from *F *had evolved higher fitness than those derived from *R *after 1000 updates (not shown). After 5 × 10^4 ^updates, however, the outcome was partially reversed, though *F *still seemed to be more evolvable than *R *for mutation rates *U *≤ 0.3 (Fig. 2A). Similar to what happened in the 8-task environment, the fitness difference between *F*- and *R*-derived lineages correlated with mutation rate (Fig. 2A; *ρ *= -0.900, 3 d.f., *P *= 0.037). Recalling that the rate of adaptation depends on the mutation rate, these results could again indicate that robustness promoted evolvability in the long-term. This was confirmed for high mutation rates (Fig. 2B), but we failed to detect such long-term benefit after 5 × 10^4 ^updates for *U *≤ 0.3, and longer times could not be explored because initial differences between *R *and *F *tended to disappear.

### General correlations between robustness and evolvability

**Figure 3:**
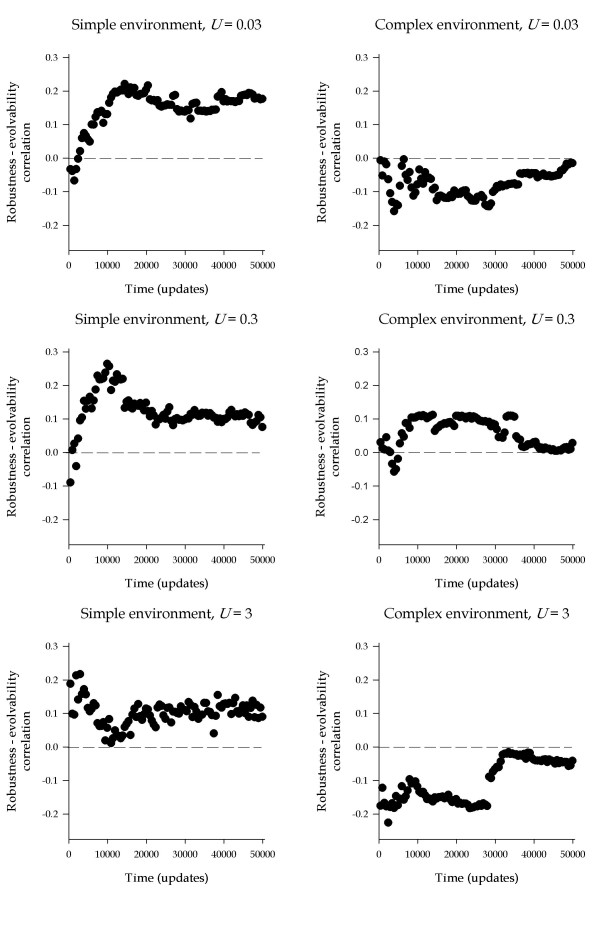
**Correlation between robustness and evolvability using all 62 genotypes obtained in the preliminary experiment. **The Spearman's correlation between the robustness of the genotype and its rate of adaptation to the two tested environments is displayed versus time. These runs were conducted at the three indicated genomic mutation rates. The null hypothesis of no correlation is indicated by the horizontal dashed lines.

To assess the generality of the above results, we analyzed the correlation between robustness and evolvability for the 62 genotypes obtained in the preliminary experiment, of which *F *and *R *were the two extreme cases. The ability of these organisms to adapt to a novel environment was assessed in the 8- and the 77-task environments at three genomic mutation rates (0.03, 0.3 and 3). In this case, only one lineage was seeded from each organism. Lineages were evolved for 5 × 10^4 ^updates and fitness values were collected every 500 updates. At each time point, a Spearman's correlation coefficient between robustness and evolvability was calculated using the 62 lineages as data points (Fig. 3).

In the 8-task environment, the results are in line with the experiments performed using the (*R*, *F*) pair. For *U *= 0.03, the correlation between robustness and evolvability remained negative during approximately the first 2500 updates and became positive from this point onwards. After update 11,500 the correlation reached a steady-state median value of 0.172, with fluctuations in the range 0.117 – 0.220 (Fig. 3). For *U *= 0.3 the correlation was null or slightly negative before update 2500, and then became positive, reaching its maximum value (0.263) around update 10^4^. From this time point on, the correlation declined until reaching a plateau value around update 15,000, and fluctuated afterwards around a median value of 0.109 (range 0.075 – 0.154). Finally, for *U *= 3, the advantage of robustness was evident right from the beginning, as supported by the positive correlation between robustness and evolvability observed along the entire experiment. In this case, the correlation fluctuated in the range 0.011 – 0.216, with a median value of 0.104. Altogether, these data are compatible with our above conclusion that robustness fosters evolvability in the long-term. The distinction between long- and short-term logically depends on the rate of adaptation which, in turn depends on the rate of mutation (see above). This is why the higher the mutation rate, the earlier the payoffs of robustness. For the three mutation rates explored, the correlation between robustness and evolvability converged to a positive value as time increased, although the exact values of these plateaus seemed to decrease as mutation rate increases.

In the more complex 77-task environment, the results were more difficult to interpret. For *U *= 0.03, the correlation between robustness and evolvability was always negative (median – 0.079; range – 0.159 to – 0.004) throughout the 5 × 10^4 ^updates of evolution, similar to the results obtained using the (*R*, *F*) pair. It is possible that the time required for robustness to confer a fitness advantage was longer than 5 × 10^4 ^updates, but after such long time the initial differences in robustness may have vanished or, alternatively, populations might have already been confined to a stable fitness value. For *U *= 0.3, robustness fostered evolvability from approximately update 5500 on, similar to what was observed in the 8-task environment. However, the benefit of robustness declined with evolutionary time, and the correlation tended to converge to zero at the end of the experiment. For *U *= 3, the correlation was always negative (median value of – 0.121; range – 0.017 to – 0.176), a result which was not consistent with the pattern observed in the 8-task environment.

## Discussion

Using the Avida digital evolution platform [26], we have investigated the effects of genetic robustness on evolvability. We first obtained organisms with varying levels of robustness by evolving a common ancestor at different combinations of mutation rates and population sizes. As previously shown [28,29], high mutation rates directly favored the evolution of robustness, whereas the effect of population size was only evident through its interplay with mutation rate. Differences in robustness evolved from populations that, apart from mutation rate, were subjected to exactly the same conditions, indicating that high mutation rates can directly select for robustness without the contribution of additional factors.

Our analyses indicate that the effect of robustness on evolvability is time-dependent, and probably also dependent on the environment and the mutation rate. In the simple environment, we observed that robustness tended to hinder evolvability in the short-term. The distinction between short- and long-term does not refer to generations but instead to stages of adaptation. As such, the effect of robustness indirectly depends on the mutation rate, since the latter affects the rate of adaptation. For low mutation rates, adaptation was slow and hence, the short-term cost of robustness endured for a relatively large number of generations. In contrast, for high mutation rates, the cost was brief or absent. We also observed that robustness consistently fostered long-term adaptation in the simple environment, a result which was weakly dependent on the mutation rate. However, for the complex environment, such a long-term benefit was less clear. The analysis of the (*R*, *F*) pair suggested that the results in the complex environment paralleled those in the simpler one, although long-term benefits occurred after larger numbers of generations. However, the analysis of the 62 genotypes obtained from the preliminary experiment indicated that the correlation between robustness and evolvability could be either positive or negative within the timeframe studied. It is possible that longer evolutionary times were required to observe a sustained positive association between robustness and evolvability. However, the genetic inheritance of robustness might be lost after such long times, due to genetic drift or to competition with fitter, less robust, genotypes.

It is noteworthy that the fraction of beneficial mutations increased with robustness, at least in the simple environment, whereas the magnitude of beneficial mutations did not significantly change. Looking at these data, one may conclude that robustness should invariantly promote evolvability, simply because beneficial mutations are more readily found. Furthermore, if beneficial mutations are rare, i.e. for short evolutionary times or for low mutation rates, the rate of adaptation ought to be mainly determined by the time required to find beneficial mutations [30], but this contradicts our observation that robust genotypes were less evolvable in the short-term, or at low mutation rates. To explain this apparent contradiction, deleterious mutations have to be incorporated into the picture. Recall that in the robust genotypes the intensity of deleterious mutational effects decreased. Given that the magnitude of fitness effects is higher on average for deleterious mutations than for beneficial ones [31], and given that the digital organisms used in our experiment reproduced asexually, the chance that a beneficial mutation was successful critically depended on whether it first appeared on a genetic background free of deleterious mutations. Owing to the weaker intensity of selection, deleterious mutations are removed less efficiently from a population of genetically robust genotypes [32] and therefore, the rate at which beneficial mutations are fixed can be slowed down in a robust genotypic background, even if such mutations appear more frequently.

Recent work has attempted to resolve the apparently paradoxical relationship between robustness and evolvability [33]. To do that, a distinction between genotypic and phenotypic robustness has been proposed. The former simply refers to the standard definition of genetic robustness and is the one used in this article, whereas the latter refers to the average genetic robustness of all genotypes encoding a phenotype. Hence, whereas genotypic robustness applies to individuals, phenotypic robustness concerns sets of neutral genotypes. If evolvability is defined as the probability of finding novel functions in the immediate mutational neighborhood (i.e. innovations involving a single mutation), genotypic robustness necessarily hampers evolvability [33]. However, phenotypic robustness would tend to promote evolvability because this kind of robustness should be associated with larger neutral network sizes, which would allow populations to explore vaster regions of the fitness landscape [33,34]. The distinction between genotypic and phenotypic robustness parallels with the distinction between short-term and long-term evolution. A founder genotype with elevated genetic robustness will perform its first adaptive steps at a slower path than a less robust one. However, as generations go on, it will degenerate on a population of genetically more heterogeneous individuals. Hence, despite decreasing phenotypic variability, robustness might foster long-term evolvability.

Two conditions have been identified as required for a system to be robust and evolvable [34]: first, genotypes need to be integrated in a highly connected neutral network, thereby implying robustness. It has been suggested that the connectedness of networks might be a very general property of evolving systems, since it has been observed independently in different processes as transcriptional regulation and RNA folding [7]. Second, neutral networks need to span a large fraction of the total genotypic space. Without this second condition, the system would be poorly evolvable, because only a small region of the fitness landscape could be explored throughout neutral mutations. In other words, the 'memory' of past robustness would be rapidly lost along evolutionary paths. This seems to be the case of RNA secondary structures, for which the effects of robustness on evolvability can only be predicted in the very short-term [33]. However, simulations suggest that neutral networks could be much more pervasive in the case of transcriptional regulation [34]. In Avida, robustness seems to be relatively stable, as suggested by the fact that initial differences in robustness were maintained after thousands of generations. In a simple environment, this stability was sufficient for the benefits of increased evolvability to be paid off before non-robust types could invade. However, we could not conclude whether the same would be true in a more complex environment. Future work will elucidate whether this condition is attainable for other evolving systems and, therefore, whether robustness can generally promote long-term evolvability.

## Conclusion

The effect of robustness on evolvability in a simple environment is time-dependent. Robustness tends to retard the first adaptive steps, whereas it fosters long-term adaptation. Furthermore, this effect also depends on mutation rate: at low mutation rates, relatively large numbers of generations are required to overcome the short-term cost of robustness. In contrast, for high mutation rates, this cost can be brief or absent. A possible explanation for these results is that deleterious mutations are removed less efficiently from a population of genetically robust genotypes, therefore interfering with the fixation of beneficial mutations. However, in the long-term, robust populations would accumulate higher levels of genetic variation and hence would be able to explore vaster regions of the fitness landscape. The complexity of the environment also plays a role in the relationship between robustness and evolvability: in a more complex environment, the correlation between robustness and evolvability could be either positive or negative within the timeframe studied.

## Methods

### The Avida platform

The Avida platform [26], was used for all experiments. In short, the genome of the starting digital organism consists of 100 instructions, with 26 possible values for each instruction [35]. Genomes are copied line by line by repeatedly executing the "copy" instruction. A subsequent execution of the "divide" instruction produces two independent genomes. Each new offspring replaces a random organism from the population, and organisms die when other organisms replace them or if 2000 instructions are executed before completing replication.

Each organism's phenotype depends upon the complex rules governing how the instructions encoded in its genome are executed. CPU time needed to execute genomic instructions is the basic resource organisms compete for. Access to CPU time depends on the metabolic rate, which was initially set to the arbitrary value 100 for each individual, but varies as organisms mutate. The investigator defines an environment by choosing a series of rewarded logical operations (e.g. AND, OR, NAND) that represent available resources. Organisms that spontaneously acquire the ability to perform these operations increase their own metabolic rate (i.e. can use more CPU time) and thus tend to increase their own replication rate. In this study, all logical resources were equally rewarded. It is important to clarify that replication rates are not user-defined but instead result from the expression of genotypes in the environment. Finally, the realized fitness of each individual depends not only on its own replication rate, but also on that of the other members of the population [26]. As in biological systems, the ability to perform tasks evolves by spontaneous mutation, selection, and drift.

Each run was initiated with a population of identical individuals. The population size was chosen by the investigator and remained constant throughout the evolution experiment (hereafter, a run). Updates are the normal time unit in Avida and represent the average time required to execute 30 instructions [26]. Here, an update corresponded to roughly 0.1 generations. Replication is subject to a fraction of point mutations, with a probability of *μ *per copied instruction or *U *per genome (*U *= 100 *μ*). The mutation rate was user-defined and constant throughout each run. In all cases, genome lengths were kept constant at 100 instructions and reproduction was strictly asexual. In any given experimental treatment, each replicate run had identical initial conditions except for a random-number seed. This initial seed causes runs to differ at all subsequent points where stochastic events occur, including mutations and the physical location where each new offspring is placed.

Experiments were performed using version 2.7.0 of Avida. This software is freely available from devolab.cse.msu.edu/software/avida. Specific details about Avida can be found elsewhere [26] and at the above website. Runs were performed using the Linux operating system on a cluster of 160 AMD Athlon 1600+ processors.

### Measures of genetic robustness and evolvability

For any given population, we first identified the most abundant genotype and then systematically constructed every possible single mutation on this genotype and tested its fitness effects, as previously described [27,28]. The signed selection coefficient of mutation *i *was expressed as *s*_*i *_= *W*_*i*_/*W*_0 _- 1, where *W*_*i *_is the fitness of the *i*^th ^mutant and *W*_0 _the fitness of the non-mutated organism. If a genotype has increased robustness, then mutations should, on average, have a little or no impact on fitness and thus, the average selection coefficient should be close to zero. By contrast, the less robust a genotype is, the more negative the average effect across all possible mutations. Genetic robustness thus can be measured as the signed average selection coefficient, excluding any possible beneficial mutations, $\bar{s_{d}}$. Finally, notice that our estimates of the average selection coefficient were obtained without error, since the fitness consequence of every possible single mutation was evaluated.

To measure evolvability we placed genotypes in a novel environment, defined by a combination of rewarded tasks (metabolic resources). The genotype was then let to evolve at a population size of 10^4^. This population size was chosen because it is large enough to allow selection to prevail over genetic drift while not compromising computational efficiency. Evolvability was measured as the observed increase in log fitness relative to the ancestor.

## Authors' contributions

SFE performed the experiments and contributed to experimental design, data analysis, and manuscript writing. RS conceived the study, contributed to experimental design and data analysis, and wrote the manuscript.
